# Assessment of Machine Learning of Breast Pathology Structures for Automated Differentiation of Breast Cancer and High-Risk Proliferative Lesions

**DOI:** 10.1001/jamanetworkopen.2019.8777

**Published:** 2019-08-09

**Authors:** Ezgi Mercan, Sachin Mehta, Jamen Bartlett, Linda G. Shapiro, Donald L. Weaver, Joann G. Elmore

**Affiliations:** 1 Paul G. Allen School of Computer Science and Engineering, University of Washington, Seattle; 2 nowwith Seattle Children’s Hospital, Seattle, Washington; 3 Department of Electrical and Computer Engineering, University of Washington, Seattle; 4 University of Vermont Medical Center, Burlington; 5 now with Southern Ohio Pathology Consultants, Cincinnati, Ohio; 6 Paul G. Allen School of Computer Science and Engineering, University of Washington, Seattle; 7 Department of Pathology and University of Vermont Cancer Center, Larner College of Medicine, University of Vermont, Burlington; 8 Division of General Internal Medicine and Health Services Research, Department of Medicine, David Geffen School of Medicine at University of California, Los Angeles

## Abstract

**Importance:**

Following recent US Food and Drug Administration approval, adoption of whole slide imaging in clinical settings may be imminent, and diagnostic accuracy, particularly among challenging breast biopsy specimens, may benefit from computerized diagnostic support tools.

**Objective:**

To develop and evaluate computer vision methods to assist pathologists in diagnosing the full spectrum of breast biopsy samples, from benign to invasive cancer.

**Design, Setting, and Participants:**

In this diagnostic study, 240 breast biopsies from Breast Cancer Surveillance Consortium registries that varied by breast density, diagnosis, patient age, and biopsy type were selected, reviewed, and categorized by 3 expert pathologists as benign, atypia, ductal carcinoma in situ (DCIS), and invasive cancer. The atypia and DCIS cases were oversampled to increase statistical power. High-resolution digital slide images were obtained, and 2 automated image features (tissue distribution feature and structure feature) were developed and evaluated according to the consensus diagnosis of the expert panel. The performance of the automated image analysis methods was compared with independent interpretations from 87 practicing US pathologists. Data analysis was performed between February 2017 and February 2019.

**Main Outcomes and Measures:**

Diagnostic accuracy defined by consensus reference standard of 3 experienced breast pathologists.

**Results:**

The accuracy of machine learning tissue distribution features, structure features, and pathologists for classification of invasive cancer vs noninvasive cancer was 0.94, 0.91, and 0.98, respectively; the accuracy of classification of atypia and DCIS vs benign tissue was 0.70, 0.70, and 0.81, respectively; and the accuracy of classification of DCIS vs atypia was 0.83, 0.85, and 0.80, respectively. The sensitivity of both machine learning features was lower than that of the pathologists for the invasive vs noninvasive classification (tissue distribution feature, 0.70; structure feature, 0.49; pathologists, 0.84) but higher for the classification of atypia and DCIS vs benign cases (tissue distribution feature, 0.79; structure feature, 0.85; pathologists, 0.72) and the classification of DCIS vs atypia (tissue distribution feature, 0.88; structure feature, 0.89; pathologists, 0.70). For the DCIS vs atypia classification, the specificity of the machine learning feature classification was similar to that of the pathologists (tissue distribution feature, 0.78; structure feature, 0.80; pathologists, 0.82).

**Conclusion and Relevance:**

The computer-based automated approach to interpreting breast pathology showed promise, especially as a diagnostic aid in differentiating DCIS from atypical hyperplasia.

## Key Points

**Question:**

Can computer vision and machine learning methods be used for automated diagnosis of preinvasive and invasive lesions of the breast to improve diagnostic accuracy?

**Findings:**

This diagnostic study of 240 breast biopsies categorized by 3 expert pathologists evaluated 2 sets of image features, which achieved sensitivity and specificity comparable with 87 pathologists in the diagnosis of breast biopsy samples. The computer-based, automated approach outperformed pathologists in differentiating ductal carcinoma in situ from atypia.

**Meaning:**

The findings suggest that machine learning methods are potentially suitable as diagnostic support systems in differentiating challenging preinvasive lesions of the breast.

## Teaser

This diagnostic study evaluates computer vision methods to assist pathologists in diagnosing breast biopsy samples, from benign to invasive cancer.

## Introduction

In 2019, more than 1 million individuals in the United States will undergo a breast biopsy, with an estimated 268 600 patients diagnosed with breast cancer and 3-fold that number receiving a noncancer diagnosis.^1^ There is growing concern in the medical community that the fear of underdiagnosing a patient leads to overdiagnosis and contributes to the ever-increasing numbers of cancer cases. Although an exact estimate of overdiagnosed cases is unknown, several studies^2,3^ have hypothesized and estimated its prevalence in breast cancer diagnosis. Whole-slide imaging (WSI), a technology that captures the contents of a glass slide in a multiresolution image, is revolutionizing diagnostic medicine by providing researchers with tools to study diagnostic missteps and develop diagnostic support systems. US Food and Drug Administration regulations limited the use of WSIs to nonclinical purposes, such as research and biorepositories, until April 2017, when the first Food and Drug Administration–approved WSI system for diagnostic medicine was announced.^4^ With this development, the US health care system will undergo a major shift toward digital pathology, and the resulting need for automated diagnosis tools that can lead to computer-aided diagnostic support systems will be significant.

Machine learning, including the use of deep neural networks, has been successfully used in a wide range of breast cancer image analysis tasks. These include differentiating between atypical ductal hyperplasia and ductal carcinoma in situ (DCIS) in mammograms,^5^ detecting lymph node metastases,^6^ automating diagnosis (including preinvasive lesions) in tissue microarray images,^7^ and identifying tumor-associated stroma in histopathology images.^8^ Automated breast cancer diagnosis has also been studied extensively in the computer vision and medical literature, yet little work exists on the full spectrum of breast lesions from benign lesions and atypia to DCIS and invasive cancer.^9,10^ Most methods deal with tumor detection only, ie, detection of invasive cancer vs noncancer,^11^ and the studies on preinvasive lesions considered the binary classification task of differentiating DCIS from benign proliferations.^10^ Invasive cancer constitutes only a quarter of the results of breast biopsies performed in the United States, while the rest are categorized into a diagnostic spectrum from benign to preinvasive disease.^12^ Preinvasive lesions, which include the categories atypia and DCIS, are associated with a higher risk of cancer, and individuals with these findings may require additional interventions and treatments. However, diagnostic disagreements are remarkably high for these preinvasive lesions. A study of 115 pathologists found that the level of agreement between pathologists and a consensus diagnosis of experts for atypia cases is only 48% (95% CI, 44%-52%).^13^ Preinvasive lesions are an integral part of the diagnostic evaluation of breast biopsy specimens and present a more difficult classification scenario for pathologists than the binary classification task of determining whether a breast biopsy sample shows invasive cancer.

We proposed novel image features for the differentiation of the full spectrum of breast lesions that covers benign, atypia, DCIS, and invasive breast cancer. In particular, we introduced the structure feature, which summarizes the architectural changes in ductal structures based on a semantic segmentation of tissue types in the breast. Our methods differ from prior work in that we attempted to emulate the behavior of pathologists as they interpret these cases by tackling successive binary decisions that were sequentially more challenging on the diagnostic difficulty scale.

## Methods

### Breast Biopsy Samples and WSIs

Digital WSIs of breast biopsies were selected from Breast Cancer Surveillance Consortium–associated tumor registries^14^ in New Hampshire and Vermont. Atypia and DCIS tend to be more challenging diagnostically; thus, these categories were oversampled relative to national estimates to increase statistical confidence for evaluating disease categories with lower prevalence. Other criteria taken into consideration during case selection included patient age and breast density. Development of the 240 cases has been previously described in detail.^15^ The hematoxylin-eosin–stained glass slides were scanned using an iScan CoreoAu scanner (Roche) (original magnification ×40). A technician and an experienced breast pathologist (D.L.W.) reviewed each digital image, rescanning as needed to obtain the highest quality. The average image size for the 240 individual WSIs was 90 000 × 70 000 pixels. Institutional review boards at the University of Washington, Dartmouth College, the University of Vermont, the Fred Hutchinson Cancer Research Center, and Providence Health and Services of Oregon approved all test set study activities.^13^

### Expert Consensus Diagnosis and Regions of Interest

A set of 14 diagnoses and 4 diagnostic categories (benign, atypia, DCIS, and invasive) was developed for the analysis. Initial diagnoses and the mapping strategy used for the final diagnostic categories are provided in eTable 1 in the Supplement. The 240 digital WSIs were interpreted independently by 3 experienced pathologists using a web-based virtual slide viewer. Each expert marked 1 or more regions of interest (ROIs) on each slide that included the features supporting their final diagnosis. Several in-person meetings and webinars were held to determine a consensus diagnosis and consensus ROIs for each digital slide, resulting in a final set of 428 ROIs including 102 benign (23.8%), 128 atypia (29.9%), 162 DCIS (37.9%), and 36 invasive ROIs (8.4%). Additional detail regarding the expert consensus review and development of the diagnostic mapping have been described.^16^ The characteristics of the 240 cases are summarized in eTable 2 in the Supplement.

As part of a larger study on diagnostic concordance in digital and traditional glass slides,^17^ pathologists from 8 US states were invited to participate in the study. Pathologists who regularly interpreted breast biopsy specimens in their clinical practices were eligible. Overall, 87 participants interpreted a subset of 60 biopsy specimens in each of the 4 subsets, with interpretations performed on the digital slides using a web-based virtual slide viewer. Each participant was randomly assigned a test set, and each participant viewed the WSIs in a randomly determined order with no time constraints. The pathologists provided informed consent. The comparison of participants’ diagnoses with expert consensus diagnoses was previously reported.^13,17^ In this work, we compare the performance of the machine learning methods on the same data set with the average performance of multiple participating pathologists who independently interpreted the same cases.

### Image Analysis

#### Overview

We used a 2-step approach, starting with semantic segmentation of the biopsy images into tissue labels, followed by feature extraction and diagnostic classification. The semantic segmentation produced a label image in which each pixel has a single tissue label. Then, the tissue label image was used to extract 2 features: a tissue distribution feature and a structure feature that is based on breast anatomy. Finally, we evaluated our features in diagnostic classification tasks.

#### Tissue Label Segmentation

**Figure 1.**
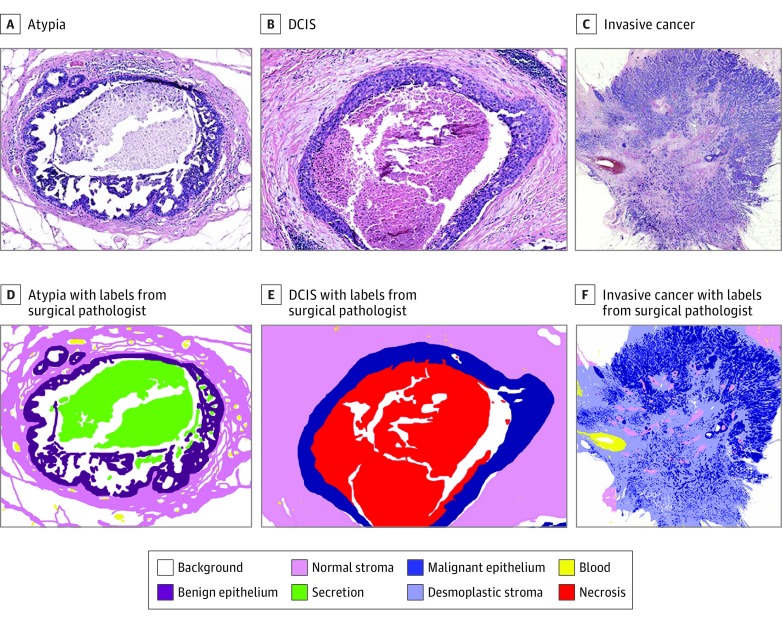
Set of Tissue Labels Used in Semantic Segmentation A-C, Unlabeled hematoxylin-eosin–stained biopsy images. D-F, Biopsy images with labels from surgical pathologists. DCIS indicates ductal carcinoma in situ.

A set of 8 clinical labels was used to annotate the breast biopsy images as follows: (1) background, (2) normal stroma (connective tissue), (3) malignant epithelium, (4) blood, (5) benign epithelium, (6) secretion (benign discharge filling the ducts), (7) desmoplastic stroma (connective tissue associated with tumor), and (8) necrosis (dead epithelial cells secondary to hypoxia) (Figure 1). Because of the expertise needed to mark the tissue labels and the size of the images, a subset of 40 specimens with 58 ROIs were randomly selected for annotation by an experienced breast pathologist (J.B.), preserving the distribution of case characteristics of the original 240 cases.

**Figure 2.**
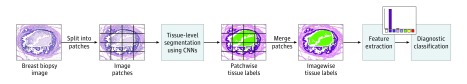
The Convolutional Neural Network (CNN) System Architecture Used for Semantic Segmentation of the Images Into 8 Tissue Labels

We implemented an end-to-end tissuewise segmentation system using a state-of-the-art convolutional neural network architecture. Our system (Figure 2) is a multiresolution encoder-decoder network with residual connections following the work of Fakhry et al^18^ that is designed to address the challenges of these images, especially the variability in size and shape of different structures present in breast biopsy images. The convolutional neural network takes input at multiple resolutions and encodes strong spatial representations by performing convolutional, downsampling, and upsampling operations. Starting with a standard encoder-decoder architecture, we used residual blocks and residual connections between encoding and corresponding decoding blocks. To aggregate features learned at different resolutions, we implemented a multiresolution network that uses a larger patch around the input image, downsamples the image to different resolutions, and applies convolutions (and deconvolutions) at different resolutions, which are again connected with residual connections. A detailed study of our segmentation system was previously described.^19^

#### Tissue Distribution Feature for Diagnosis

A basic visual difference between diagnostic categories in pathology is the existence (and amount) of different biological structures. Using the simple linear iterative clustering^20^ algorithm, the images were segmented into superpixels*,* which are regions of similar color with an area of about 3000 pixels. The size and other parameters of the superpixel segmentation were selected so that a superpixel covered at least 1 epithelial cell. Each superpixel was then assigned a tissue label (the label belonging to most of its pixels) based on the label image produced in the previous step. To capture the distribution and simple spatial relationships of the tissues, we calculated the tissue distribution by means of frequency and co-occurrence histograms over superpixels.

#### Structure Feature for Diagnosis

**Figure 3.**
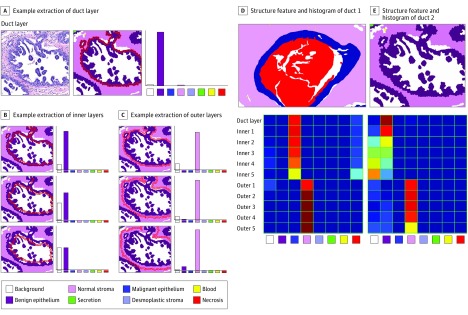
Example Structure Features Starting with the tissue label segmentation, epithelium labels are used as the object of interest. The superpixels at the border of the duct are used to construct the first histogram for the duct layer, in which red indicates the highest value and dark blue the lowest. The same process was repeated for 5 inner and 5 outer layers of the duct. The superpixels belonging to a layer are marked with red borders. A, Photomicrograph shows hematoxylin-eosin–stained biopsy image.

Our structure feature describes the changes in the shape of the epithelial structures in the breast biopsy slides. Using the epithelium labels assigned by the semantic segmentation, we identified objects of interest, which may be a duct, a group of ducts, or a tumor. Then, starting from the outer border of the object, we extracted 5 layers toward the inside of the object and 5 layers toward the outside. For each layer, we calculated a normalized frequency histogram of 8 tissue types. Figure 3A-C shows the calculation of the structure feature for a duct. Figure 3D-E illustrates the structure feature for 2 different duct images whose labels are shown as pseudocolors above and whose structure feature histograms are shown below. In the histograms, the columns represent the 8 labels, and the rows represent the 10 layers. The histogram bin values are shown as a heat map in which red is the highest value and dark blue is the lowest.

In our implementation, we used superpixels as the structural elements. The layers of the structure feature were defined 1-superpixel thick, and the layer histograms were built by counting superpixels. Although other structural elements can be used (eg, patches, pixels, or hexagons), superpixels provide a good definition for the object borders and are widely used in segmentation.^21^

The definition of a layer and the number of layers can be adapted for different data sets and different problems. In our implementation, we defined the layers starting from the outer border of the objects of interest, ie, ducts. We used 5 inner and 5 outer layers. Because the size of the superpixels was selected based on the size of an average epithelial cell in our images, the first 1 or 2 layers of epithelial superpixels at the circumference of the duct would define a normal duct. For our data set of breast biopsy images, 5 inner layers and 5 outer layers were generalizable to all diagnostic categories yet still powerful enough to describe the structural changes.

**Figure 4.**
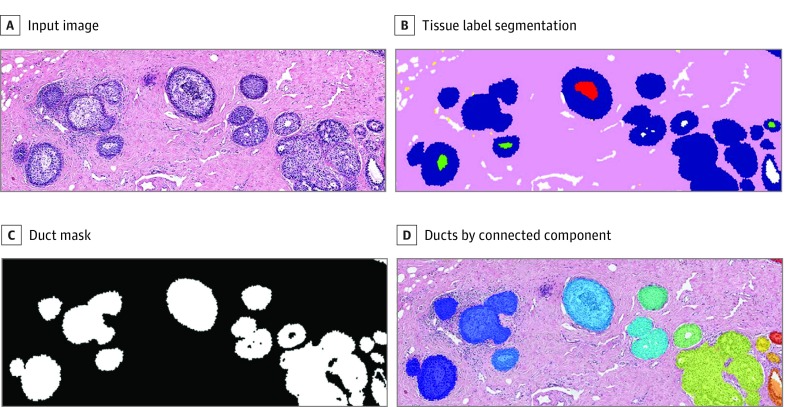
Preprocessing to Detect Ducts as Objects of Interest for the Structure Feature A, Input image shows hematoxylin-eosin–stained biopsy image.

A critical step in the implementation of the structure feature is the definition and detection of the objects of interest, ie, ducts. Breast ducts are composed of epithelial cells; however, in certain cases, ducts were filled with secretion or necrosis. To get a complete picture of the structure, we used epithelium (both benign and malignant), secretion, and necrosis labels to obtain a binary image. After cleaning small objects, we applied connected components analysis to identify individual objects (ducts or duct groups). Figure 4 shows an example image with its tissue label segmentation, the binary image of the union of epithelium, secretion, and necrosis labels, and the detected objects overlaid on the original image.

The structure features were calculated for the ducts, but the diagnostic classes were assigned to the ROIs. To obtain a feature vector for each image, the histograms for each layer were summed up. Ideally, we would have liked to classify each duct. Although the experts marked the smallest possible ROI, some still contained benign ducts or lesser diagnoses than the ROI assigned. Although we averaged the structural properties of all the ducts in an ROI by summing the histograms, the feature vector should be dominated by the largest structures in the ROI.

#### Diagnostic Classification

The diagnostic decision-making process, during which pathologists interpret the whole slide at the resolution at which different image characteristics are best viewed, is complex. For example, the high-level organization of tissue that is needed to diagnose invasive cancer is available to the observer at lower resolutions, whereas the structural and cellular features that distinguish preinvasive lesions are usually observed at higher resolutions. Furthermore, the features that describe an invasive carcinoma do not apply to other diagnoses; in other words, different visual cues are used for different diagnoses. It may not be reasonable to expect a machine algorithm to accurately classify an image de novo into the full range of diagnostic categories. Based on this observation and discussions with expert pathologists and practicing clinicians on how a clinical diagnosis is performed, we designed a classification scheme in which a decision is made for a single diagnosis at a time, as follows: (1) classification of invasive cancer or noninvasive diagnosis, (2) classification of noninvasive diagnosis into preinvasive lesions (atypia and DCIS) or benign, and (3) classification of DCIS or atypia.

We evaluated the performance of the tissue distribution feature and the structure feature. Whole slide images can contain multiple diagnoses, but the ROIs were carefully marked by our clinical experts to represent the final diagnosis. Therefore, we used the 428 ROIs with the consensus diagnosis labels as our data set in diagnostic classification experiments.

For all experiments, we trained and tested support vector machine classifiers with a third degree polynomial kernel in a leave-1-out cross-validation setting. When the sample size was smaller than the number of features, we applied principal components analysis and used the first 20 principal components to reduce the number of features. During training, we subsampled the training data to have an equal number of samples for each class so that the random chance for classifying each slide to a diagnostic category was 50% and the trained machine learning model had no bias toward the larger diagnostic class. To reduce the effect of subsampling, we repeated all experiments 100 times and reported the average accuracies.

### Statistical Analysis

We reported classification accuracies as well as sensitivity and specificity metrics for the classification experiments. In terms of true positives (TPs), true negatives (TNs), false-positives (FPs), and false-negatives (FNs), accuracy was defined as (TP + TN) / (TP + TN + FP + FN). Sensitivity was defined as TP / (TP + FN), and specificity was defined as TN / (TN + FP). We also reported the accuracy (correct classification rate) for each classification task. Cross-validation experiments were repeated 100 times with subsampling, and accuracies were reported. All models were developed and trained in MATLAB (R) (Mathworks) using the LIBSVM open-source library.^22^

## Results

The Table shows the results of the automated analyses on the 3 tasks and for different features compared with the expert consensus reference standard for assessing accuracy. The comparison is between which feature was used (ie, tissue distribution feature or structure feature). In addition, the results of the automated analyses on each of the 3 tasks are shown compared with the results of the US pathologists who interpreted these samples in the digital WSI format. As there were 87 pathologists distributed to 1 of 4 test sets, each specimen was reviewed by 18 to 24 practicing pathologists.

**Table.** Performance of Machine Learning Image Features for Diagnostic Classification Compared With Diagnoses of 87 Practicing US Pathologists Who Independently Interpreted the Same Cases

Diagnostic Feature	Sensitivity	Specificity	Accuracy^a^
**Invasive vs Noninvasive**			
Tissue distribution feature	0.70	0.95	0.94
Structure feature	0.49	0.96	0.91
Pathologists	0.84	0.99	0.98
**Atypia and DCIS vs Benign**			
Tissue distribution feature^b^	0.79	0.41	0.70
Structure feature^b^	0.85	0.45	0.70
Pathologists	0.72	0.62	0.81
**DCIS vs Atypia**			
Tissue distribution feature	0.88	0.78	0.83
Structure feature	0.89	0.80	0.85
Pathologists	0.70	0.82	0.80

Abbreviation: DCIS, ductal carcinoma in situ.

^a^ Accuracy, also called the correct classification rate, does not necessarily provide information on accuracy in clinical practice as the composition of test cases does not represent the prevalence of disease found in the general population.

^b^ Uses support vector machine–based segmentation instead of convolutional neural network.

In the classification of invasive cases, the tissue distribution feature achieved the highest accuracy (0.94). The same feature had a low sensitivity (0.70) but a high specificity (0.95). In other words, 30% of the invasive cases were missed, but there were very few FPs. In comparison, the participants had a 0.84 sensitivity and 0.99 specificity.

The most difficult task was the separation of benign samples from atypia and DCIS. We achieved only 0.70 accuracy using the structure feature with the support vector machine–based segmentation. The same feature had a sensitivity of 0.85 and a specificity 0.45. In other words, almost half the benign cases were overdiagnosed as atypia or DCIS, but the FN rates were low. In comparison, the participants had 0.72 sensitivity and 0.62 specificity.

The classification of DCIS and atypia with the structure feature achieved 0.85 accuracy, 0.89 sensitivity, and 0.80 specificity. In comparison, the participants had 0.70 sensitivity and 0.82 specificity for the DCIS cases.

## Discussion

We proposed a novel machine learning approach, the structure feature, for the classification of breast tissue. Our experiments showed that when there is no defined ductal structure, such as in invasive cancer, a tissue distribution feature, which captures the frequency and co-occurrence of the tissues, is sufficient for classification. In other words, simpler features may be considered for mass screenings that identify invasive carcinomas, and more sophisticated features, such as our structure feature, may be considered for a finer subclassification of preinvasive ductal lesions. Because invasive cancer does not follow the rules and principles of normal tissue organization, it is logical to remove invasive cancer from the larger pool of specimens prior to classification of the remaining high-risk lesions, which we accomplished with our stepped approach to classification.

Early work in this field by Mercan et al^23,24^ used an older class structure of 5 breast tissue classes. The ROI-level classification results obtained an accuracy of 0.77, but the sensitivity was only 0.42 with specificity of 0.85. In other words, their classifier was very good at finding TNs, but it was not very good at detecting cancer. The ensuing work by Gecer et al,^25^ which also used the 5-class diagnostic structure, obtained an overall accuracy of 0.55 with their method of majority voting and saliency detection, but they did not report sensitivity and specificity. Mehta et al^26^ developed an entirely new deep learning architecture called Y-net for simultaneous segmentation and classification at the ROI-level. Using the 4-class structure that is defined in the present article, they obtained an overall accuracy of 0.63 and did not report on sensitivity and specificity.^26^ A major emphasis of this paper by Mehta et al^26^ was the structure and speed of the deep neural network.

The work in our study expands beyond these articles, as we have designed a different method for classification, using a hierarchical structure instead of a single 4-way or 5-way classifier. Our approach mimics the diagnostic decision-making process of a pathologist who eliminates a single diagnosis at a time instead of choosing between multiple diagnoses. Our results also show improved accuracy compared with these earlier works. In clinical practice, data on sensitivity and specificity are important and useful parameters. The overall accuracy of a research test set may be influenced by the distribution of cases (eg, the test set used in earlier work had a higher prevalence of cases of atypia and DCIS, thus making the overall accuracy lower than expected in clinical practice). We therefore presented data on sensitivity and specificity for each step in our work. In the classification tasks of atypia and DCIS vs benign and DCIS vs atypia, the associated sensitivities are higher than the sensitivity of the practicing pathologists who independently interpreted the same specimens.

### Strengths and Limitations

The strengths of our study include a large set of images that comprised a full spectrum of diagnostic categories and a novel approach to a difficult diagnostic classification problem. Although larger WSI data sets exist, they almost always lack the diagnostic diversity or image quality of our data set. Instead of approaching this topic as a multiclass classification, we ran multiple experiments to determine which features and approach work best for differentiating a range of diagnoses. An important aspect of our work is that we also compared our results with interpretations on each case from a large pool of actively practicing pathologists with varying experience in breast pathology. Our ground truth segmentation data was also provided by a surgical pathologist who spent months carefully painting the labels on a set of training data.

While machine learning methods hold great promise in the field of pathology, we recognize the limitations of our study. In clinical practice, a diagnosis is rendered with multiple cross-sections and images rather than a single image per patient, as provided in our study. Additional clinical information and second opinions are also available to pathologists, which may improve their accuracy in a real-life clinical setting. Furthermore, we expect that the finer classification of epithelial atypia will require more annotation work but may improve the performance of diagnostic classification. Additionally, our system works on manually marked regions of interest, but with the use of an ROI-detection system,^25,27^ it could be extended to WSIs.

## Conclusions

This study presents a novel machine learning feature, called the *structure feature*, for describing the area around a duct in WSIs of breast biopsies. Using a data set of 240 breast biopsy samples ranging from benign epithelial proliferation to invasive carcinoma, we conducted a thorough set of experiments with multiple methods of tissue segmentation and diagnostic classification and compared the machine learning results with interpretations from a group of practicing pathologists. The best classification results came close to those of the pathologists for invasive vs noninvasive diagnoses, lagged behind them in diagnosing atypia and DCIS vs benign specimens (which the pathologists also found difficult), and outperformed them in differentiating DCIS from atypia, which is considered the most diagnostically difficult task in clinical practice. With further improvements in the machine learning techniques we used—or even improvements at the cellular level—it may be possible for computer vision methods to be used in conjunction with pathologists’ intuition to improve diagnostic performance.

## Notes

Supplement. **eTable 1.** Hierarchical Description Showing the Mapping Used to Characterize Individual Interpretations Into 1 of the 4 Major Categories Used in this Analysis **eTable 2.** Patient and Case Characteristics of 240 Whole Slide Images
