# Supplementary material for: Assessment of Machine Learning of Breast Pathology Structures for Automated Differentiation of Breast Cancer and High-Risk Proliferative Lesions
Source: JAMA Netw Open. 2019 Aug 9;2(8):e198777. doi: 10.1001/jamanetworkopen.2019.8777 (PMC6692690; doi:10.1001/jamanetworkopen.2019.8777)
Supplement: Supplement. — eTable 1. Hierarchical Description Showing the Mapping Used to Characterize Individual Interpretations Into 1 of the 4 Major Categories Used in this Analysis eTable 2. Patient and Case Characteristics of 240 Whole Slide Images [file jamanetwopen-2-e198777-s001.pdf]

Supplementary Online Content

Mercan E, Mehta S, Bartlett J, Shapiro LG, Weaver DL, Elmore JG. Assessment of machine learning of breast pathology structures for automated differentiation of breast cancer and high-risk proliferative lesions. *JAMA Netw Open*. 2019;2(8):e198777. doi:10.1001/jamanetworkopen.2019.8777

eTable 1. Hierarchical Description Showing the Mapping Used to Characterize Individual Interpretations Into 1 of the 4 Major Categories Used in this Analysis
eTable 2. Patient and Case Characteristics of 240 Whole Slide Images

This supplementary material has been provided by the authors to give readers additional information about their work.

© 2019 Mercan E et al. *JAMA Network Open.*

eTable 1. Hierarchical Description Showing the Mapping Used to Characterize Individual Interpretations Into 1 of the 4 Major Categories Used in this Analysis

Diagnosis	Mapping
Non-proliferative Changes Only	Benign
Fibroadenoma (FA)[b]	Benign
Intraductal Papilloma Without Atypia	Benign
Usual Ductal Hyperplasia (UDH)	Benign
Columnar Cell Hyperplasia / Columnar Cell Change	Benign
Sclerosing Adenosis	Benign
Radial Scar/ Complex Sclerosing Lesion	Benign
Flat Epithelial Atypia (FEA)[c]	Atypia
Atypical Ductal Hyperplasia (ADH)	Atypia
Intraductal Papilloma With Atypia	Atypia
Atypical Lobular Hyperplasia (ALH)[d]	Atypia
Ductal Carcinoma in-situ (DCIS)	DCIS
Lobular Carcinoma in-situ (LCIS)	DCIS
Invasive (ductal or lobular) cancer	Invasive

[a]Only data from the Phase II digital arm is included in this analysis. Please refer to: Elmore JG, Longton GM, Pepe MS, et al. A Randomized Study Comparing Digital Imaging to Traditional Glass Slide Microscopy for Breast Biopsy and Cancer Diagnosis. J Pathol Inform. 2017;8:12. Published 2017 Mar 10. doi:10.4103/2153-3539.201920. In this previous paper, data were reported on N=86 pathologists who were randomized to the digital format in Phase II. The current paper includes data from one additional pathologist who requested to interpret cases in digital format in Phase II, thus N=87).
[b] FA is grouped with Benign. FA is technically a proliferative lesion but has little associated risk.
[c] FEA was grouped with ADH in the atypia category because FEA may lead to excision in some institutions.
[d] ALH is grouped with ADH in the atypia category and LCIS is grouped with DCIS following traditional cancer progression schemes.

© 2019 Mercan E et al. *JAMA Network Open.*

eTable 2. Patient and Case Characteristics of 240 Whole Slide Images

Patient and case characteristics	# Cases (%)
**Patient characteristics**	
Age	
40–49 years	118 (49.2)
50–59	122 (50.8)
Breast density	
Almost entirely fat	13 (5.4)
Scattered fibroglandular densities	105 (43.8)
Heterogeneously dense	97 (40.4)
Extremely dense	25 (10.4)
**Case characteristics**	
Biopsy type	
Core needle biopsy	138 (57.5)
Excisional biopsy	102 (42.5)
Expert consensus diagnosis*	
Benign	72 (30.0)
Atypia	72 (30.0)
DCIS	73 (30.4)
Invasive cancer	23 (9.6)
**Total**	**240 (100.0)**

- The expert consensus diagnosis was obtained using original glass slides, after independent interpretation by three experienced breast pathologists followed by in person meetings using a modified Delfi method to define the expert consensus diagnosis on each case.

© 2019 Mercan E et al. *JAMA Network Open.*
